# Homologous repair deficiency score for identifying breast cancers with defective DNA damage response

**DOI:** 10.1038/s41598-020-68176-y

**Published:** 2020-07-27

**Authors:** Ahrum Min, Kwangsoo Kim, Kyeonghun Jeong, Seongmin Choi, Seongyeong Kim, Koung Jin Suh, Kyung-Hun Lee, Sun Kim, Seock-Ah Im

**Affiliations:** 1 Biomedical Research Institute, Seoul National University Hospital, Seoul, Republic of Korea; 2 Cancer Research Institute, Seoul National University, Seoul, Republic of Korea; 3 Department of Internal Medicine, Seoul National University Bundang Hospital, Seoul, Republic of Korea; 4 Department of Internal Medicine, Seoul National University Hospital, Seoul, Republic of Korea; 5 Department of Computer Science and Engineering, Seoul National University, Seoul, Republic of Korea; 6 Interdisciplinary Program in Bioinformatics, Seoul National University, Seoul, Republic of Korea; 7 Bioinformatics Institute, Seoul National University, Seoul, Republic of Korea; 8 Translational Medicine, Seoul National University College of Medicine, Seoul, Republic of Korea

**Keywords:** Cancer, Genetics

## Abstract

Breast cancer (BC) in patients with germline mutations of *BRCA1/BRCA2* are associated with benefit from drugs targeting DNA damage response (DDR), but they account for only 5–7% of overall breast cancer. To define the characteristics of these tumors and also to identify tumors without BRCA mutation but with homologous recombination deficiency (HRD) is clinically relevant. To define characteristic features of HRD tumors and analyze the correlations between *BRCA1/BRCA2* and BC subtypes, we analyzed 981 breast tumors from the TCGA database using the signature analyzer. The BRCA signature was strongly associated with the HRD score top 10% (score ≥ 57) population. This population showed a high level of mutations in DDR genes, including *BRCA1/BRCA2*. HRD tumors were associated with high expression levels of *BARD1* and *BRIP1*. Besides, *BRCA1*/2 mutations were dominantly observed in basal and luminal subtypes, respectively. A comparison of HRD features in BC revealed that *BRCA1* exerts a stronger influence inducing HRD features than *BRCA2* does. It reveals genetic differences between *BRCA1* and *BRCA2* and provides a basis for the identification of HRD and other BRCA-associated tumors.

## Introduction

Depending on hormone receptor and human epidermal growth factor type II receptor (HER2) oncoprotein expression, breast cancer (BC) is traditionally classified into luminal A or B (i.e., estrogen and/or progesterone receptor-positive), HER2-enriched, or triple-negative BC (TNBC). However, there is high heterogeneity, even within subtypes, making treatment difficult^1,2^. To improve treatment, understanding tumor heterogeneity within and across subtypes and proper treatment strategies for each tumor is crucial. One approach is examining the mutations of each tumor and defining distinct molecular signatures for categorization. The Cancer Genome Project (TCGA) serves as an important basis for understanding the genomics of tumor heterogeneity^2^.

Poly (ADP-ribose) polymerase (PARP) inhibitors are clinically beneficial in patients with BRCA-deficient ovarian, breast, and prostate cancer. In a global phase III trial involving patients with metastatic BC with germline *BRCA1* or *BRCA2* mutation, olaparib increased progression-free survival (PFS) by 2.8 months^3^. In an olaparib maintenance clinical phase II trial of patients with relapsed platinum-sensitive ovarian cancer in addition to BRCA-deficient tumors, one-third of patients with wild-type BRCA showed improvements in PFS^4,5^. Based on this, olaparib was additionally approved by the FDA for maintenance treatment in platinum-sensitive patients, regardless of BRCA status. These results suggest that BRCA and other homologous repair deficiency (HRD) markers determine response to PARP inhibitors and that these should be applied to patients with other HRD markers as well. In fact, tumors lacking BRCA mutation but with HRD, similar responses to DNA damaging agents, and similar clinicopathologic features are referred to as having “BRCAness,” and the use of PARP inhibitors has been expanded to these tumors. The Sanger Institute group suggested that mutational signatures 3 and 8, identified based on analysis of deletion or substitution patterns through microhomology, can best predict HRD indicating BRCAness^6,7^. Although mutational signatures can predict BRCAness, factors that induce BRCAness remain unclear, and no markers have been suggested for > 30% of cases of BRCAness.

A genome study of ovarian cancer recently revealed that *BRCA1* and *BRCA2* deficiency, either of which is considered the most important risk factor for hereditary BC and ovarian cancer, lead to different structural changes and mutation patterns in tumor genomes. *BRCA1* variations cause greater genomic instability than *BRCA2* variations in ovarian cancer^8^, and ovarian cancer patients with *BRCA2* germline mutations show better clinical outcomes than *BRCA1*-mutated patients^9,10^. However, the genetic features resulting from *BRCA1* or *BRCA2* deficiency in BC have not been compared, and a single *BRCA1/2*-deficient BC subtype is typically considered. Furthermore, based on a previous finding that the BRCA-deficient BC subtype is highly associated with TNBC, medications such as PARP inhibitors targeting BRCA-associated cancers have been used in limited cases, such as BRCA variations and the TNBC subtype^11^. However, while *BRCA1* deficiency is highly correlated with estrogen receptor-negative BC, no correlation has been reported between *BRCA2* deficiency and the incidence of any specific BC subtype^11^.

Here, we used the TCGA database of 981 patients with BC to evaluate screening methods that better reflect *BRCA* germline mutations or characteristics of BRCAness and to identify genetic alterations within BRCA-associated cancer. This study presents a guideline for BRCA-associated cancer-targeted therapy by analysing correlations between *BRCA1/2* and different BC subtypes. The study defines features useful for identifying *BRCA1*- or *BRCA2*-deficient cancer types regardless of subtype.

## Results

### Correlation between BC subtype and specific mutational signatures

**Figure 1:**
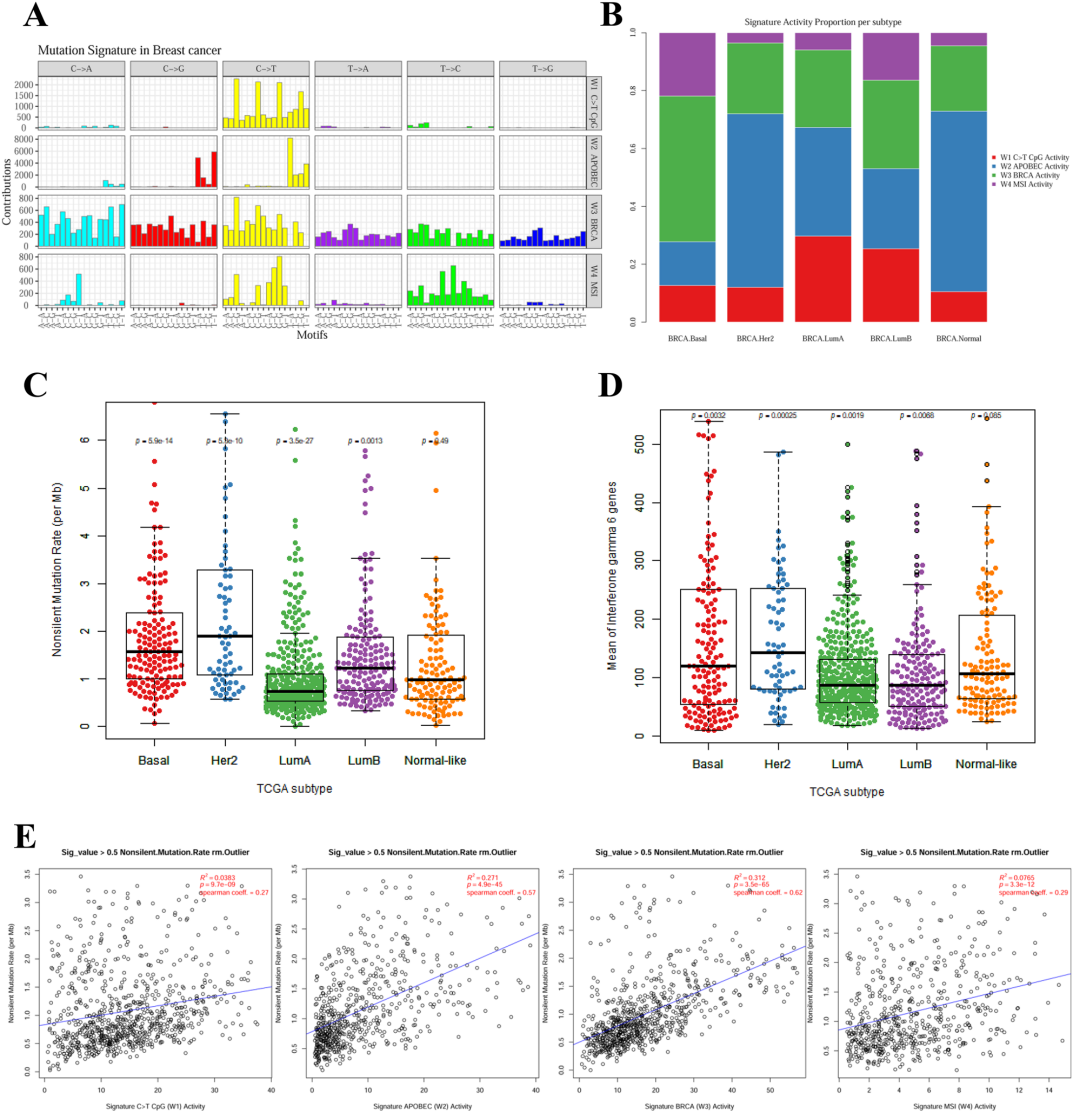
Characterization of four mutational signatures. (**A**) Characterization of four distinct mutational signatures. Trinucleotide 96 plot for the finally selected mutational signatures. Different colours represent one of the six base substitutions, and each base substitution is categorized according to the 5′ and 3′ nucleotides. Each signature is named based on the closest Cosmic signature. W1 sig. is C > T CpG signature (cosmic 1) with cosine similarity 0.94; W2 sig. is APOBEC signature (cosmic 2 + 13) with cosine similarity 0.85 (cosmic 2) and 0.81 (cosmic 13); W3 sig. is BRCA signature (cosmic 3) with cosine similarity 0.86; W4 sig. is MSI signature (cosmic 6) with cosine similarity 0.83. (**B**) The relationship between subtype and mutational signatures. For each PAM50 subtype, the distribution of first signature activity is expressed as a bar graph. (**C**) The non-silent mutation rate based on PAM50 subtype. (**D**) The relationship between subtype and IFN-γ signature. IFN-γ signature was estimated by the man of the expression of six genes (IDO-1, CXCL10, CXCL9, HLA-DRA, STAT1, IFNG). (**C**,**D**) The central line in the box plot represents the median. Wilcoxon rank-sum test *p*-value was calculated based on one versus rest. (**E**) The relationship between mutation burden and signature. The scatter plot shows only the samples with signature activity ≥ 0.5, after removing the outlier based on the Tukey rules. R^2^ and p refer to the R-squared value and the *p*-value of the linear model, and the blue line represents the regression trend line. Tukey's rule is as follows: For first quartile Q1, second quartile Q2, third quartile Q3, and IQR = Q3–Q1; samples smaller than Q1–1.5IQR or larger than Q3 + 1.5IQR are regarded as outliers and removed.

We classified somatic mutation patterns in 981 individual BC tissues into mutational signatures using the non-negative matrix factorization (NMF) technique and characterized the activities and types of signatures for each tumor using *Signature analyzer*^12^. Two cases, one in which a signature was observed in a single case only and one case with a high single-nucleotide variation (SNV) rate of ≥ 5,800, were excluded. Four signatures were identified in the remaining 981 tumor tissues after 50 rounds of Bayes NMF (Fig. 1A) and were highly consistent with those already reported^12^: the APOBEC signature (COSMIC 2 and 13), C > T CpG signature (COSMIC 1A), MSI signature (COSMIC 6), and BRCA signature (COSMIC 3), all of which exhibited a COSMIC signature with cosine similarity ≥ 0.83 (Supplementary Fig. S1). The BRCA, APOBEC, and CpG signatures were prominent in the basal-like, HER2-enriched, and luminal BC subtypes, respectively (Fig. 1B). To explore the possibility of using immune checkpoint inhibitors in the subgroups, we also analysed the mutation burden of each tumor^13–15^. The mutation burden was higher in the basal-like, HER2-enriched, and luminal B subtypes (Fig. 1C). Moreover, the mutation burden according to subtype was correlated with the IFN-γ signature defined with 6 immune-related genes, a predictor of the effectiveness of immune checkpoint inhibitors (Fig. 1D). The mutation burden was also associated with the mutational signature, as the BRCA signature correlated with a high mutation burden (Fig. 1E). Along with the observations that cytotoxic T-lymphocyte antigen 4 (CTLA4), programmed cell death ligand-1 (PD-L1), and indolamine 2,3-dioxygenase 1 (IDO1) expression levels were upregulated in signature 3 tumors and that BRCA-mutated BCs showed increased tumor infiltrating lymphocytes (TILs) compared with BRCA wild-type, these data suggest the potential of using immune checkpoint inhibitors in BRCA-signature tumors^16,17^.

### HRD scores as predictors of BRCA germline mutations and “BRCAness”

**Figure 2:**
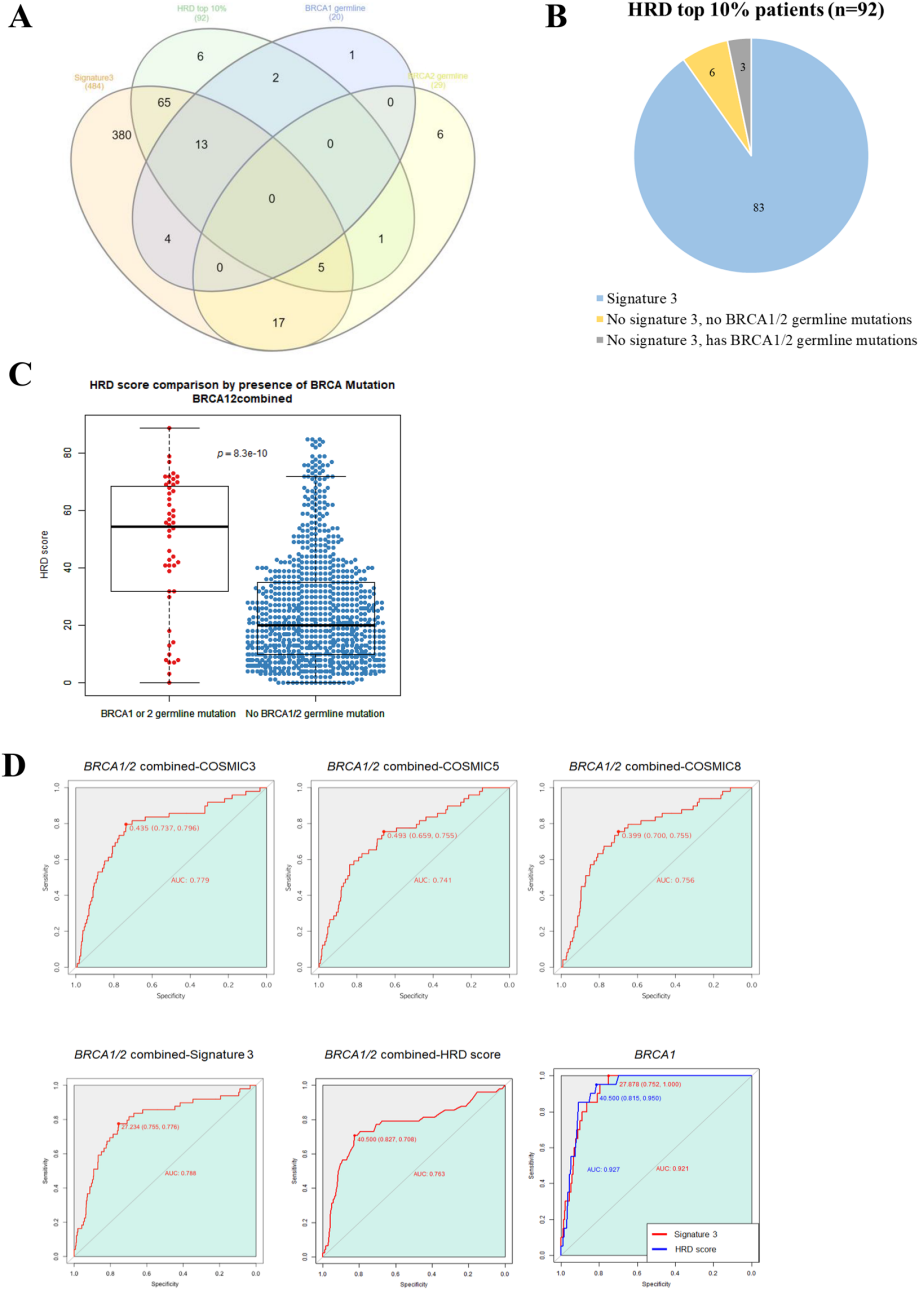
The association with HRD and signature 3. (**A**) Signature 3 determined by the *Signature analyzer*, and the relationship between the tumors in the top 10% of HRD scores (HRD tumors) and tumors with *BRCA1* or *BRCA2* germline mutation. Most patients in the top 10% HRD, *BRCA1* germline, and *BRCA2* germline group were included in the Signature 3 group. Signature 3: Patients whose *Signature analyzer*-derived signature 3-like value was the most dominant (n = 484); HRD top 10%: Patients with HRD scores in the top 10% (n = 92), *BRCA1* germline: Patients who had a *BRCA1* germline mutation (n = 20); *BRCA2* germline: Patients who had a *BRCA2* germline mutation (n = 29). (**B**) The inclusion relationship between HRD tumors and Signature 3 population. Among the tumors with a top 10% HRD score, six patients had no *BRCA1/2* germline mutations, while only three had none. (**C**) The difference in HRD score between samples with *BRCA1* or *BRCA2* germline mutation and those without mutation. Wilcoxon rank-sum test produced the *p*-value = 8.3e−10. The central line in the box plot represents the median. (**D**) Assessment of the prediction accuracy among different variables with respect to *BRCA1* and *BRCA2* germline mutation. The maximum sum between sensitivity and specificity is indicated.

Although a significant correlation has been reported between signature 3 and *BRCA1/2* germline mutations, the optimal cut-off for selecting tumors with mutational signature 3 activity is unclear^12^. Furthermore, some tumors with a BRCA-related signature (signature 3) do not harbour mutations in *BRCA1/2* or other homologous recombination (HR) pathway genes, making it difficult to define signature 3 tumors as HRD tumors. We compared various methods for identifying tumors with “BRCAness”. *Signature analyzer* identified signature 3 as the 1^st^ signature in 49.33% (n = 484) of all tumors in this study^12^. However, we assumed that not all of these exhibited “BRCAness”^18,19^. Thus, we identified 86 cases that exhibited 1^st^ cosine similarity with COSMIC signature 3 or 8, because these are related to “BRCAness” or HRD (Supplementary Fig. S2). As HRD scores are considered biomarkers of genomic instability with mutation, we analysed the correlation between tumors identified as signature 3 by *Signature analyzer* and those with HRD scores in the top 10% (≥ 57; n = 92), assuming that high HRD scores are reflective of HR-deficient tumors, like those with BRCA mutations. Only nine tumors within the top 10% of HRD scores failed to show an association with signature 3, and three of those had *BRCA1/2* germline mutations (Fig. 2A,B). To investigate how well the top 10% of HRD scores reflect HRD, we evaluated how well the scores predicted *BRCA1/2* germline mutations, which are well-known contributors to HRD. Comparisons of the HRD scores in the presence (median 54.5) and absence (median 20) of BRCA germline mutations indicated a ~ two fold increase in the score when germline mutations were present (Fig. 2C). In addition, the area under the curve (AUC) for detecting germline mutations of *BRCA1* based on the top 10% of HRD scores was 0.927, and the AUC for *BRCA1/2* was 0.763. Therefore, HRD scores in the top 10% may be useful predictors of *BRCA1/2* germline mutations, especially for *BRCA1* (Fig. 2D). Previous reports have suggested that the RPS, which is based on the expression of four genes involved in DNA repair pathway preference, can also be used to predict sensitivity to DNA-damaging agents and HRD^20^. Thus, we evaluated how well the RPS predicted HRD. A significant correlation was found between the RPS and the top 10% of HRD scores or *BRCA1* and/or *BRCA2* germline mutations (mean RPS, with *BRCA1* germline mutation: − 98.00926; with *BRCA2* germline mutation: − 89.43415; with *BRCA1/2* germline mutations: − 92.6938; with top 10% HRD: − 91.78309; and with bottom 90% HRD: − 84.12268; Supplementary Fig. S3). These findings suggest that the RPS could be another useful tool for identifying “BRCAness”.

### Alterations in DDR genes in HRD tumors determined by HRD scores

**Figure 3:**
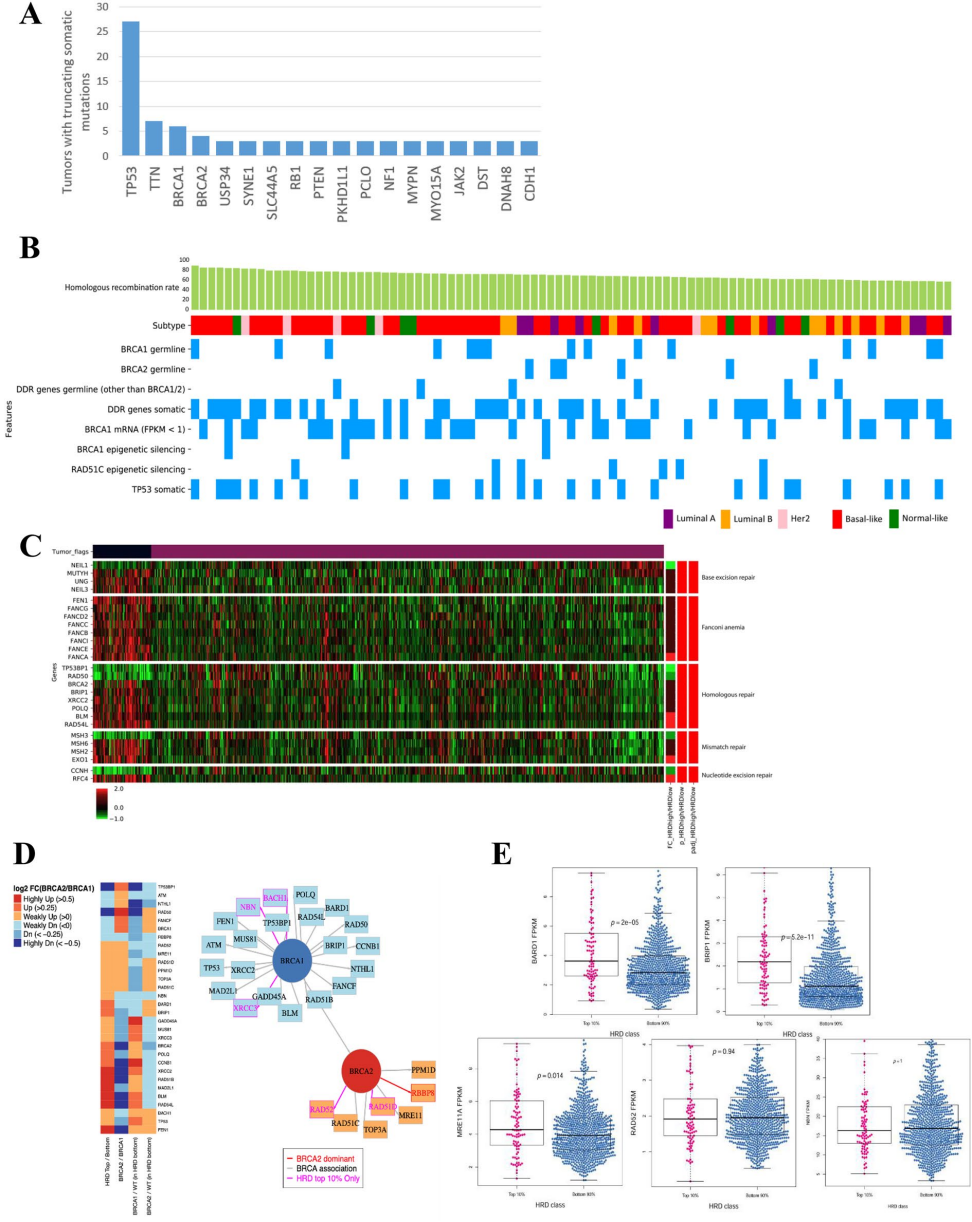
Identifying the genetic features that determine HRD tumors. (**A**) The frequency of somatic alterations in tumors among the top 10% of HRD scores (HRD tumors). Genes were sorted by their truncating mutation count within HRD tumor patient group. The bar graph presents the list of genes with truncated somatic alterations in more than three tumors. (**B**) Genetic alterations in HRD tumor population. Of the 92 HRD tumors, 9 cases with *BRCA1* germline mutations and 2 cases with *BRCA*2 germline mutations exhibited somatic alterations in DDR genes, and among these tumors, *TP53* alteration was observed in 9 cases. Among the tumors with germline mutations in *BRCA1* or *BRCA2*, there were two cases with an additional *POLQ* germline alteration and one case with a *BRIP1* germline mutation. Subtype colour codes are as follows. Purple: luminal A, orange: luminal B, pink: Her2, red: basal-like, green: normal-like. (**C**) Differences in DDR pathway-associated expression observed in HRD tumors. Among the 88 DDR genes (redundant genes excluded), we observed a significant expression difference in genes in the HR, BER, and FA pathways. Expression values are colour-coded for each gene, the lowest expression value being bright green, the middle being black, and the highest being bright red. Tumor flags: Patients with HRD tumors are labelled black, and patients with non-HRD tumors are labelled maroon. Fold change: HRD tumor expression mean was divided by that of non-HRD tumors, and then a log2 transformation was applied, followed by colour-coding. Deep red indicates a high positive log2 fold change, and deep blue indicates a high negative log2 fold change; *p*-value: Differences in expression among groups were compared using the rank-sum test. Genes that had a nominal *p*-value < 0.05 were labelled red, and the others white; Adjusted *p*-value : For all nominal *p*-values, multiple testing correction was performed by adjusting the *p*-values using the default *p*.adjust function in R. (**D**) *BRCA1* plays a more dominant role than *BRCA2* in promoting development into HRD tumors. When the expression levels of the 30 genes involved in the HR pathway were compared for each group (HRD tumors vs. non-HRD tumors; *BRCA2* germline mutation vs. *BRCA1* germline mutation; *BRCA1* germline mutation non-HRD tumors vs. *BRCA1* WT non-HRD tumors; *BRCA2* germline mutation non-HRD tumors vs. *BRCA2* WT non-HRD tumors), 23 genes showed an identical pattern to that in *BRCA1* germline mutation HRD tumors, while 7 genes showed an identical pattern to that in *BRCA2* germline mutation tumors, and 5 genes showed an expression pattern specific to HRD tumors, irrespective of BRCA germline mutation. (**E**) High *BARD1* and *BRIP1* expression in HRD tumors. Comparing the expression levels of the 5 genes that showed a difference between HRD and non-HRD tumors showed significantly high levels for *BARD1* (*p* = 5.2e−11) and *BRIP1* (*p* = 2e−05) in HRD tumors. For calculating the *p*-value, the Wilcoxon rank-sum test was used, and the central line in the box plot represents the median.

The median HRD score was 20 for tumors without BRCA germline mutations and 54.5 for tumors with BRCA germline mutations. The median HRD score for the overall population of 981 was 21 (Fig. 2C). Based on this finding, tumors with HRD scores ≥ 57, corresponding to the top 10%, were defined as HRD tumors. These tumors were highly associated with the COSMIC 3 and 5 signatures, which are BRCA-associated signatures (Supplementary Fig. S4)^6^. To assess the characteristics of HRD tumors, we assessed somatic truncation alterations in DDR genes frequently observed in such tumors. The most frequently observed alteration was in *TP53* (n = 27), followed by *TTN*, *BRCA1* and *BRCA2* (Fig. 3A). Up to three alterations were observed in DDR genes within a single tumor, and this tumor also exhibited a *BRCA1* germline mutation (p.C61G). Of the 92 HRD tumors, 9 with *BRCA1* germline mutations and 2 with *BRCA*2 germline mutations exhibited somatic alterations in DDR genes, and among these, *TP53* mutation was observed in 9 cases (Fig. 3B and Supplementary Fig. S5). In addition, 81 out of 981 tumors had germline mutations in one or more of the 11 DDR genes (including *BRCA1/2*). Among tumors with germline mutations in *BRCA1/2*, there were two cases with an additional *POLQ* germline alteration and one with a *BRIP1* germline mutation. These genes were exclusively mutated in 78 cases with germline mutation in DDR genes (Supplementary Fig. S6). In the study by Polak et al., which used the same dataset to define the characteristics of the population with signature 3 activity in the top quarter, the authors reported a correlation between germline mutations in *PALB2* and signature 3 activity in two cases^12^. However, in the HRD tumor population analysed in this study, no germline alteration in *PALB2* was observed. After analysing the genetic alterations in HRD tumors, we identified 57 cases with germline or somatic mutations in 44 DDR-associated genes, three with *BRCA1* epigenetic silencing, and seven with *RAD51C* epigenetic silencing (Fig. 3B and Supplementary Fig. S5). Specifically, 36 cases out of the HRD tumor population exhibited low levels of *BRCA1* mRNA. Among these, eight exhibited germline mutations in *BRCA1* and three had hypermethylation of the promoter (Fig. 3B and Supplementary Fig. S5). The remaining cases had suppressed transcription regardless of promoter methylation. To understand whether HRD tumors affect the activation of specific DDR pathways, we classified the DDR pathways into nine types and selected genes that acted exclusively on each pathway to compare their expression levels (Supplementary Table S1). Among the HRD tumors, a significant difference in the expression levels of these genes was observed among the HR repair pathway, Fanconi anaemia (FA) pathway, and base excision repair (BER) pathway (Fig. 3C). Interestingly, in the HRD tumors, genes involved in DDR pathways exhibited relatively high expression. Notably, the expression of core genes in the HR pathway (including *BRIP1, BLM, POLQ, RAD54L,* and *FANCA*) was upregulated, and that of *NEIL1* in the BER pathway and *TP53BP1* and *RAD50* in the HR pathway was significantly downregulated (Fig. 3C). In contrast, the expression of *BLM*, *FEN1*, and *BRCA2* (direct transcriptional targets of *BRCA1* that are negatively regulated) was upregulated in HRD tumors^21,22^, as was expression of *EXO1*, *NEIL3*, and *BRIP*^23^. The protein expression of FANC family members was also upregulated. Interestingly, in HRD tumors, expression of *BRCA2* was relatively high, while that of *BRCA1* was low. These results suggest different patterns of expression between *BRCA1*-associated tumors and *BRCA2*-associated tumors. Then, we compared the expression of the 30 genes involved in the HR pathway between HRD and non-HRD tumors, between tumors with *BRCA1* germline mutations and those with *BRCA2* germline mutations, and between those with wild-type *BRCA1* and *BRCA2* and non-HRD tumors (Fig. 3D). *NBN, BARD1,* and *BRIP1* are associated with *BRCA1*, and *MRE11* and *RAD52* are associated with *BRCA2*; their expression was upregulated only in HRD tumors^24–27^. When differences in expression between HRD and non-HRD tumors were examined, regardless of the presence or absence of *BRCA1/2* mutations, only *BARD1* and *BRIP1* showed significantly higher expression levels in HRD tumors (Fig. 3E), indicating that expression of *BARD1* and *BRIP1* may predict HRD tumors. These findings suggest that in BC, *BRCA1* mutation results in higher HRD scores than *BRCA2* mutation, further supporting the existence of differences between *BRCA1* and *BRCA2* germline mutations in the induction of genomic instability.

### *BRCA1* and *BRCA2* germline mutations exhibit different features in BC

**Figure 4:**
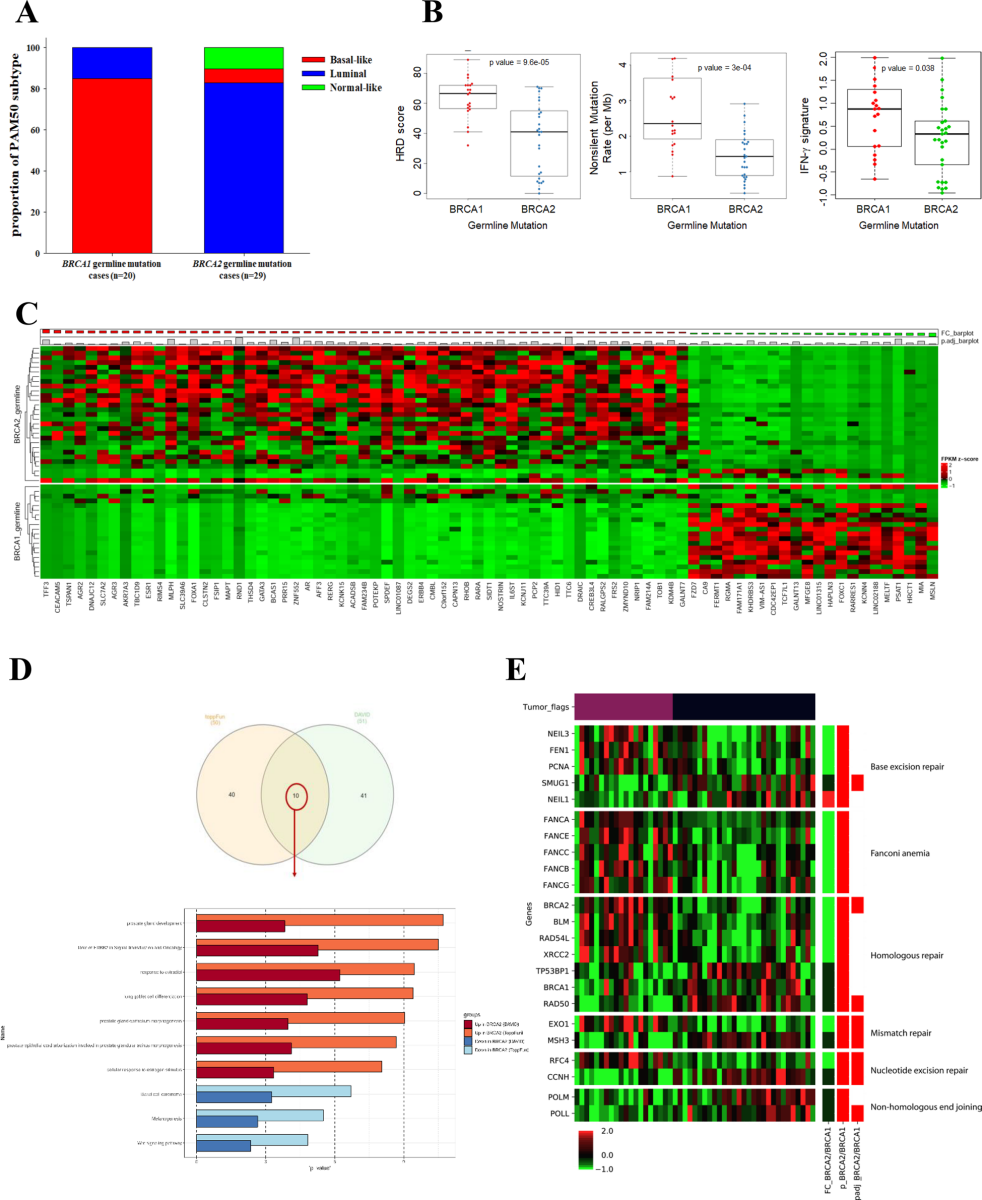
Distinct characteristics between *BRCA1* and *BRCA2* germline mutated tumors. (**A**) Difference in subtype distribution. For the PAM50 subtype proportion and subtype colour of 20 tumors with *BRCA1* germline mutation and 29 tumors with *BRCA2* germline mutation, red represents basal-like, blue represents luminal (A + B), and green represents normal-like. (**B**) Difference in genomic instability pattern depending on BRCA germline mutation. In each group with *BRCA1* and *BRCA2* germline mutation, the extent of HRD score, the non-silent mutation rate, and IFN-γ signature were compared. In all cases, significantly higher levels were observed in the case of *BRCA1* germline mutation. For calculating the *p*-value, the Wilcoxon rank-sum test was used, and the central line in the box plot represents the median. (**C**) Heat map showing gene expression in *BRCA1* germline mutants and *BRCA2* germline mutants. Based on the FPKM value for the genes, the intergroup fold change (FC = mean FPKM of *BRCA2* germline mutants/mean FPKM of *BRCA1* germline mutants) was calculated, and the genes with abs(log2(FC)) > 1.5 and Wilcoxon rank-sum test/Benjamini & Hochberg adjusted p-value < 0.05 were selected. The plotting was based on the column (gene) z-scale of log2FC values. (**D**) Activation of different functional pathways in cancer depending on *BRCA1/2* germline mutation. Functional annotation analysis was conducted for the 79 genes that exhibited gene expression between the two groups of *BRCA1* germline mutation and *BRCA2* germline mutation, and *p*-value plot was used to visualize the ten commonly obtained functional pathways from the two tools. (**E**) Difference in DDR gene expression patterns for *BRCA1* germline mutation and *BRCA2* germline mutation groups. Among the 88 genes, those that exhibited an intergroup difference were grouped according to the pathway, and a significant expression difference in genes in the HR, BER, and FA pathways was observed. Expression values are colour-coded for each gene, the lowest expression value being bright green, the middle being black, and the highest being bright red. Tumor flags: Patients with *BRCA1* hotspot or truncating germline mutation are labelled maroon, and patients with *BRCA2* hotspot or truncating germline mutation are labelled black; Fold change: *BRCA2*-mutated group expression mean is divided by the *BRCA1*-mutated group, and then a log2 transformation is applied and then colour-coded. Deep red indicates a high positive log2 fold change, and deep blue indicates a high negative log2 fold change; *p*-value: Differences in expression among groups were compared using the rank-sum test. Genes that had a nominal *p*-value < 0.05 were labelled red, and the others white. Adjusted *p*-value: For all nominal *p*-values, multiple testing correction was performed by adjusting the *p*-values using the default *p*.adjust function in R.

We also investigated subtypes of breast cancer induced by *BRCA1/2* germline mutations, as well as their different characteristics. Forty-nine cases had germline mutations in *BRCA1* or *BRCA2*. Of these, one with both *BRCA1* and *BRCA2* mutation was excluded. More than 85% of tumors with *BRCA1* germline mutations were the basal-like subtype, whereas 83% with *BRCA2* germline mutations were the luminal subtype (Fig. 4A). The extent to which *BRCA1/2* mutations induce genomic instability also significantly differed; tumors with *BRCA1* mutations showed significantly higher HRD scores and mutation burden than those with *BRCA2* mutations. Corresponding to these results, the median expression of IFN-γ signature genes showed higher in tumors with *BRCA1* germline mutations than *BRCA2* germline mutations (*p* = 0.038; Fig. 4B). Moreover, we identified 79 genes that were differentially expressed in the tissues of BC patients with germline *BRCA1* and *BRCA2* mutations (Fig. 4C). For these DEGs, a functional annotation analysis was conducted using two tools: DAVID and ToppFun. Genes with increased expression from both tools in common were involved in the Wnt signalling pathway. Those upregulated in tumors with germline *BRCA1* mutation were related to endothelial-to-mesenchymal transition (EMT) in basal cell carcinoma. For tumors with germline *BRCA2* mutation, upregulated DEGs were involved in HER2 signal transduction and in estrogen signalling (Fig. 4D). These results imply that the mechanisms of carcinogenesis differ between tumors with germline *BRCA1* and *BRCA2* mutations. Thus, to investigate how defects in *BRCA1* and *BRCA2* differentially influence the DDR pathway, differences in the expression levels of 88 genes involved in DDR pathways were assessed. The expression of genes involved in the HR pathway was significantly upregulated in *BRCA1*-deficient tumors compared with levels in *BRCA2*-deficient tumors (Fig. 4E). The presence of *BRCA1* germline alteration is more closely involved in HRD than that of *BRCA2*, and it is anticipated that BRCA-associated cancer may be differentiated even in the absence of *BRCA1* germline mutation if a similar expression profile is exhibited. These results also suggest that differential therapeutic strategies could be effective for *BRCA1*-deficient and *BRCA2*-deficient tumors. For example, combining hormone or HER2-targeted therapies with DNA damage-inducing agents such as PARP inhibitors could be investigated in *BRCA2*-deficient tumors, and combining immune checkpoint inhibitors or inhibitors of EMT might be effective for *BRCA1*-deficient tumors.

## Discussion

PARP inhibitors, such as olaparib or talazoparib, are effective in BC patients with germline *BRCA1/2* mutations^3,28^. Approximately 7% of patients with BC and 11–15% of TNBC patients harbour germline *BRCA1/2* mutations. In this study, including somatic alterations, 10% of the 981 BC patients exhibited either *BRCA1* or *BRCA2* mutation^12^. To identify patients who could potentially benefit from PARP inhibitors or other DDR-inducing drugs beyond those with *BRCA1/2* mutation is a relevant clinical topic, and the concept of “BRCAness” has been raised. These include tumors associated with genetic alterations in *RAD51C*, *PALB2*, *BARD1*, *RAD51D*, and *CHEK2*, and recent studies report that HRD could be caused by germline mutations in *ATM*, *BAP1*, *CDK12*, and *FANCM*^29^. With a growing emphasis on HRD in cancer treatment, efforts have been made to define the HRD phenotype. Large-scale genome analysis of 10,952 exomes and 1,048 whole genomes in 40 human cancer types (including BC) was performed, and 30 mutational signatures were defined^30^. From analysis of the whole-genome sequencing data of 560 BC tissues, 12 mutational subtypes were identified, 5 of which were further clustered into those with APOBEC deamination and those with mismatch repair-deficiency, based on the characteristics of their DNA replication strand biases. Additionally, tumors with germline mutations in *BRCA1* and *BRCA2* possess characteristic mutational patterns, such as signature 3 and signature 8^19^. Signature 3 is characterized by large insertions and deletions at breakpoint junctions and is strongly associated with germline mutations in *BRCA1* and *BRCA2*. Because patients showing signature 3 are highly responsive to platinum-based chemotherapy, it is believed that their HR repair is defective^31,32^ However, a study conducted by Nik-Zainal et al. on TCGA data from 560 patients with BC revealed that 50% of the patients showing a signature 3 pattern had BRCA mutations, whereas the remaining 50% had dysfunctional BRCA-related DNA repair, despite the absence of BRCA mutations^19^. Such tumor cases were classified as having “BRCAness”. Thus, in addition to BRCA genes, markers that induce features such as BRCAness have drawn attention. In a 2017 study of genetic alterations in 995 TCGA BC cases, 247 cases were in the top quartile of signature 3 activity^12^. Of those, 88 showed biallelic loss in *BRCA1* or *BRCA2*, whereas the remaining 159 patients were classified as having BRCAness. Among the 10% with the most BRCAness, 2% showed epigenetic silencing of *RAD51C* and germline mutations in *PALB2* and *BARD1*. The study revealed that signature 3 selectively reflects BRCA germline mutations and BRCAness and thus has the potential to be useful for treatment strategies targeting BRCA-associated BC^12^. However, the factors that induce BRCAness remain unclear, and no markers have been suggested for > 30% of cases of BRCAness. HRD score provides a method to analyse loss of heterozygosity (LOH), telomeric allelic imbalance (TAI), and large-scale state transition, thereby better reflecting chromosomal instability. In a large phase III trial of niraparib, a PARP inhibitor, in ovarian cancer, neither the LOH-based HRD score nor Foundation Medicine T5 NGS assay could discriminate the responders^7^. The study defined HRD tumors based on an HRD score ≥ 42, accounting for approximately the top 20% of HRD scores in the TCGA data. Considering that an HRD score of 48.5 provides the most plausible prediction of *BRCA1* germline mutation, it is presumed that their failure might have been in setting the cut-off so low, resulting in an ambiguous definition of HRD tumors. In fact, analysis of the receiver operating characteristic (ROC) curve showed that, for an HRD score of 42, the maximum sum value of specificity was 0.8, suggesting that the cut-off was not suitable for distinguishing between the HRD and HR-proficient populations (data not shown). In this study, ROC curve analysis was used to confirm that the HRD cut-off was above a score of 48.5, which best reflects *BRCA1* germline mutation (Supplementary Fig. S7a). Moreover, the population was selected such that the score that predicts both *BRCA1/2* germline mutations would effectively have a maximum sum value of specificity ≥ 0.9. Tumors with HRD scores in the top 10% were highly associated with BRCA-associated signatures, such as COSMIC 3, 5, and 8 signatures, while those with HRD scores ≥ 42 (the cut-off used in previous studies) were associated with both the BRCA-associated signature and CpG signatures (Supplementary Fig. S7b). An HRD score ≥ 57 accounts for 10% of the total population, and the specificity of 0.920045 is anticipated to be suitable for actual HRD tumor screening.

Active research on markers that predict the effects of immune checkpoint inhibitors have made claims that tumors with severe genomic instability are the most responsive. In addition, numerous next-generation sequencing studies have reported that, in mutational signature 3-associated tumors with an HRD phenotype, expression *CTLA-4*, *PD-L1*, and *IDO-1* is elevated^16,33^. In fact, cases with *BRCA1/2* germline mutation with high genomic instability show significantly higher expression levels of immunogenic lymphocytes and TILs than those without these mutations, lending further support to the close association between genomic instability and immunogenic features^34^. Based on these findings, a phase II study is underway for the single-agent therapy of pembrolizumab*,* a PD-L1 inhibitor, in BC patients with BRCA germline mutation (NCT03025035). In the current study, a strong IFN-γ signature was observed for the basal and HER2-enriched subtypes with high mutation burden, and a positive correlation between signature 3 activity and mutation burden was verified. Based on this, therapeutic effects of immune checkpoint inhibitors may be anticipated in BC with high genomic instability. Furthermore, beneficial effects may be expected after extending the therapeutic target to HRD tumors with signature 3 in BC patients with BRCA germline mutation. This also provides an adequate rationale for trials involving combination therapy of a PARP inhibitor that is known to cause genomic instability in BC and a PD-1 or PD-L1 inhibitor as an immune checkpoint inhibitor, currently in phase I/II (NCT02484404, NCT03594396, and NCT03167619).

Despite continued efforts to locate major genetic alterations that cause HRD besides the BRCA germline mutations, describing HRD in terms of germline and somatic alterations in DDR genes has been considerably limited^12,35,36^. The current study focused on identifying characteristic expression patterns of genes in HRD tumors. As a result, we found two genes, *BRIP1* and *BARD1*, that are involved in the HR pathway and show high expression only in the HRD tumor population. Both genes are well-known *BRCA1*-associated genes. BARD1 is widely recognized as an essential partner for the function of BRCA1, as it participates in cytokinesis and the regulation of DNA repair by forming a complex with BRCA1 to regulate topoisomerase IIa activity and regulates ubiquitin ligase activity for the ubiquitination of histone protein and RNA polymerase II^24,37,38^. Moreover, *BARD1* knockout models display almost an identical phenotype to *BRCA1* knockout models^39^. However, BARD1 is also reported to be involved in BRCA1-independent oncogenic signalling modulation to facilitate cancer progression. Further, it is reported to increase sensitivity to PARP inhibitors based on the HRD phenotype caused by high expression of *BARD1 SV* and *BARD1β*, the oncogenic isoforms of *BARD1* in colon cancer^40,41^. *BARD1* expression in breast and ovarian cancer also shows positive correlations with poor prognosis^42^. A role for BRIP1 in DNA repair has been reported, as it forms a complex with BARD1 by interacting with BRCA1 as a DNA helicase^25,43^. In addition, germline mutation of *BRIP1* is associated with HRD. However, a recent study reported that, although the germline mutation of *BRIP1* is a strong risk factor in ovarian cancer, it is difficult to view it as a significant factor in BC^44,45^. In contrast to its mutation, high expression of *BRIP1* is reported to correlate with poor prognosis, based on which it was anticipated that *BARD1* and *BRIP1* would prove to be significant HRD markers in BC^46,47^. Furthermore, during the search for *BRCA1/2*-associated factors, the current study identified *RBBP8* (CtIP), whose expression level was reduced specifically in *BRCA2* mutation-associated HRD tumors. A recent study reported that, while *RBBP8* and *BRCA1* disruption led to a marked increase in chromosomal instability, there was no significant influence on chromosomal stability when both *BRCA2* and *RBBP8* were deficient, and that the formation of a complex between CtIP and BRCA1 played a role in *MRE11* maintenance^48,49^. These findings suggest that defects in *BRCA1* and *RBBP8* are mutually exclusive. Moreover, a high expression level of *RBBP8* is maintained in hormone-positive BC, and reduced *RBBP8* expression is a mechanism of resistance to tamoxifen^50,51^. These findings predict that *RBBP8* may have a role in the transcription and signalling regulation of hormone genes, which may rely on a *BRCA1*-dependent mechanism.

Based on the current study, we propose that the HRD score provides a more effective means to define true HRD tumors in BC than the conventional method of identification using COSMIC signature and *Signature analyzer*. Furthermore, through this study, we suggest that *BARD1* and *BRIP1*, which are upregulated in HRD tumors, may act as novel HRD markers. Lastly, our study revealed that tumors with germline *BRCA1* and *BRCA2* mutations showed different genetic and biological features, inducing differences in the expression of genes involved in hormone signalling and Wnt signalling. Furthermore, *BRCA1* mutation was found to have a stronger influence than *BRCA2* mutation on chromosomal structure and mutation induction, as well as on HRD induction via DDR gene expression regulation. Based on our findings, *BRCA1*-associated regulation is thought to be a more potent HRD-inducing factor in BC, and the *BRCA1* and *BRCA2* defects should be understood as having distinct features.

## Materials and methods

### Dataset

Whole-exome sequencing and transcriptome data from 992 patients with BC were downloaded from TCGA. Data from 981 patients for whom signature analysis could be performed were used. Clinicopathological characteristics are outlined in Table 1. All mutation, clinicopathological, RNA-Seq, methylation sequencing, PAM50, and HRD score data were obtained from TCGA.

**Table 1:** Descriptive information of TCGA dataset.

Characteristics	Number of patients (%)
**Sex**	
Female	970 (98.9)
Male	11 (1.1)
**TNM stage**	
I	167 (17.0)
II	567 (57.8)
III	216 (22.0)
IV	21 (2.1)
N/A	10 (1.0)
**Estrogen receptor status**	
Positive	718 (73.2)
Indeterminate	2 (0.2)
Negative	216 (22.0)
N/A	45 (4.6)
**PAM50 subtype**	
Luminal A	443 (45.2)
Luminal B	179 (18.2)
HER2	70 (7.1)
Basal-like	154 (15.7)
Normal-like	122 (12.4)
N/A	13 (1.3)
**Germline BRCA mutation**	
*BRCA1*	20 (2.0)
*BRCA2*	30 (3.0)
*BRCA1* and *BRCA2*	1 (0.1)
**Signature**	
CpG	316 (32.2)
APOBEC	161 (16.4)
BRCA	484 (49.3)
MSI	20 (2.0)

### Germline mutation calling

VarScan2 was used with the following parameters to call germline variants from tumor/normal pair alignment files from TCGA: VarScan2 somatic, —min-coverage 30, —min-var-freq 0.08, —normal-purity 1, —*p*-value 0.10, —somatic-*p*-value 0.001, and —output-vcf 1^52^. The somatic option was used to allow VarScan2 to annotate germline and somatic variants using tumor and normal alignment files as input. ANNOVAR was used to annotate variant function^53^. In-house filters were applied to remove: (1) low quality germline variants (depth < 10 or variant count < 5 or variant allele fraction < 0.08 or labelled “somatic” by VarScan2), (2) variants near homopolymer regions, (3) variants with absolute difference in mapping quality ≥ 30 between reference and variant reads, (4) variants positioned in read ends, and (5) common variants with a minor allele frequency ≥ 1%, as annotated in dbSNP and gnomAD. Only variants affecting protein function were selected by removing those annotated as being in intergenic, intronic, untranslated region (UTR), upstream, downstream, or noncoding RNA (ncRNA) regions. To select variants likely to disable protein functions, only truncating variants were selected by filtering out all mutation types other than known hotspot mutations annotated in ClinVar, frameshift indels, splice site mutations, and nonsense substitutions. Germline variants in *ATM, BAP1, BRCA1, BRCA2, BRIP1, CDK12, CHEK2, NBN, PALB2, POLQ,* or *RAD51C* were extracted. A flowchart of DNA damage response (DDR) gene germline mutation calling is provided in Supplementary Fig. S8.

### Mutation signature analysis

TCGA mutation annotation format (MAF) data were compiled into four file types depending on the variant caller used: Mutect^54^, MuSE^55^, SomaticSniper^56^, and Varscan^52^. The files included only mutations detected by at least two or more variant callers, and the variants of all 981 patients were detected by ≥ 2 variant callers. Data were analysed using the *Signature analyzer* program of the Broad Institute.

At the 50^th^ iteration, five signatures were obtained from over 40 iterations: the W1 signature showed a Cosine similarity of 0.94 with COSMIC1; W2 showed a Cosine similarity of 0.82 with COSMIC6; W3 showed a Cosine similarity of 0.97 with COSMIC10; W4 showed a Cosine similarity of 0.85 with COSMIC2; and W5 showed a Cosine similarity of 0.86 with COSMIC3. Up to 69% of W3 was occupied by a single sample (TCGA-AN-A046); thus, it was determined to be a singleton signature caused by an ultra-mutant tumor, and TCGA-AN-A046 was excluded from the subsequent round of *Signature analyzer*. At the 50^th^ iteration, four signatures (S4) were obtained from over 40 iterations, while five signatures (S5) were obtained from fewer than 10 iterations. S4 and S5 exhibited two marked differences: (1) C > T APOBEC (COSMIC2) and C > G APOBEC (COSMIC13) were separated in S5, but conjoined in S4; (2) the similarity with COSMIC3 was 0.8 for S5, but 0.86 for S4. Thus, S4 was chosen based on its higher level of similarity with COSMIC and the robust result from *Signature analyzer*. The data are presented in Supplementary Table S2.

### Deconstruct sigs

The R package *deconstructSigs* was used to identify mutational signatures of samples. TCGA MAF provided single-nucleotide polymorphisms (SNPs) in BC samples, which were converted to a 96-trinucleotide matrix categorizing 96 base substitutions according to the nucleotides positioned 5′ and 3′ of the corresponding SNP. The mutational signature contribution of each sample was calculated using the *whichSignatures* function of *deconstructSigs*, and the COSMIC signature was used as a reference.

### Differential expression and pathway enrichment analysis

Differentially expressed genes (DEGs) were defined as those with an absolute log2 fold change between the average fragments per kilobase per million mapped reads (FPKM) for each gene of *BRCA2* germline mutants and that for each gene of *BRCA1* germline mutants > 1.5 and with a false discovery rate (FDR) ≤ 0.05 estimated using the Benjamini & Hochberg method. Functional annotation analysis was conducted using two DAVID version 6.8 and ToppFun, after dividing the DEGs into 57 and 22 genes that were upregulated in the *BRCA2* and *BRCA1* germline, respectively^57^. DAVID was used to determine Gene Ontology (GO) and pathways, while ToppFun was used to analyse GO terms (molecular function, biological process, cellular component) and pathways under the following conditions: *p*-value method was the probability density function; FDR correction and *p*-value cut-off were set to 0.05; gene limits were set to 1 ≤ n ≤ 2,000. The intersection between the results produced by the two tools displayed ten terms, and they were visualized as a *p*-value plot.

### HRD score and mutation rate

For the HRD, silent mutation rate, and non-silent mutation rate, the published dataset based on the TCGA pan-cancer database was used^58^. The top 10% and bottom 90% of HRDs were differentiated based on 936 TCGA BC samples: the top 10% (n = 92) had HRD values ≥ 57; the bottom 90% (n = 844) had HRD values ≤ 56.

### Recombination proficiency score (RPS)

RPS was calculated from the equations given below, using the FPKM of the microarray provided by the TCGA^20^.

$${\text{RPS}}\, = \,{-}1{\text{ }} \times {\text{ }}\left( {RIF1 + PARPBP + RAD51 + XRCC5} \right)$$

$$\begin{aligned} {\text{RPS}}\,{\text{beta}}\, = \, & {-}1\, \times \,\left[ {\left( {0.2171423{\text{ }} \times RIF1} \right){\text{ }} + {\text{ }}\left( {0.1946173{\text{ }} \times PARPBP} \right) + \left( {0.2783017{\text{ }} \times RAD51} \right)} \right. \\ & \quad \quad \left. { + {\text{ }}\left( {0.3099387{\text{ }} \times XRCC5} \right)} \right] \\ \end{aligned}$$

### Epigenetic silencing

To determine the methylation status of 12 DDR genes, the sum of the methylation levels of the promoter region was calculated using 450 K methylation data from the PanCancer Atlas, with a methylation value of ≥ 0.75 defined as hypermethylation. Among cases of hypermethylation, those with coupled mRNA levels of < 1 FPKM were defined as exhibiting “epigenetic silencing,” as the transcription of the gene was suppressed by the epigenetic event of DNA hypermethylation.

### Statistics

The Wilcoxon rank-sum test was used to assess differences between two groups. For three or more groups, One versus Rest was applied, and then the Wilcoxon rank-sum test was used. To analyse pathway activation with intergroup differences, the adjusted *p*-value (FDR) was calculated using the Benjamini and Hochberg method following the Wilcoxon rank-sum test based on the *wilcox.test* function in R.

## Supplementary information

Supplementary file1 (PDF 1121 kb)
