# Supplementary material for: Homologous repair deficiency score for identifying breast cancers with defective DNA damage response
Source: Sci Rep. 2020 Jul 27;10:12506. doi: 10.1038/s41598-020-68176-y (PMC7385153; doi:10.1038/s41598-020-68176-y)
Supplement: Supplementary file 1 — Supplementary file1 (PDF 1121 kb) [file 41598_2020_68176_MOESM1_ESM.pdf]

Supplementary Information

Homologous Repair Deficiency score for identifying breast cancers with defective DNA damage response

Running head: Homologous repair deficiency in breast cancer

Ahrum Min[1,2,*], Kwangsoo Kim[1,*], Kyeonghun Jeong[1], Seongmin Choi[1], Seongyeong Kim[2], Koung Jin Suh[2,3], Kyung-Hun Lee[2,4,†], Sun Kim[5,6,7], and Seock-Ah Im[1,2,4,8]†

[1] Biomedical Research Institute, Seoul National University Hospital, Seoul, Korea

[2] Cancer Research Institute, Seoul National University, Seoul, Korea

[3] Department of Internal Medicine, Seoul National University Bundang Hospital, Seoul, Korea

[4] Department of Internal Medicine, Seoul National University Hospital, Seoul, Korea

[5] Department of Computer Science and Engineering, Seoul National University, Seoul, Korea

[6] Interdisciplinary Program in Bioinformatics, Seoul National University, Seoul, Korea

[7] Bioinformatics Institute, Seoul National University, Seoul, Republic of Korea

[8] Translational Medicine, Seoul National University College of Medicine, Seoul, Korea

*These authors contributed equally to this work.

† To whom correspondence should be addressed: Seock-Ah Im, Tel: 82-2-2072-0850; Fax: 82-2-762-9662; E-mail: moisa@snu.ac.kr. Kyung-Hun Lee, Tel: +82-2-2072-7207; Fax: +82-2-762-9662; E-mail: kyunghunlee@snu.ac.kr

Supplementary Table S1. List of the key genes for each DNA repair pathway

Fanconi Anemia	Non-homologous end joining	nucleotide excision repair	base excision repair	translesion synthesis	homologous recombination	mismatch repair	direct repair
FANCA	*LIG4*	*CCNH*	*APEX1*	*MAD2L2*	*ATM*	*EXO1*	*MGMT*
FANCB	*LIG4*	*CDK7*	*APEX2*	*REV1*	*BARD1*	*MLH1*	
FANCC	*POLL*	*DDB1*	*Fen1*		*BLM*	*MLH3*	
FANCD2	*POLM*	*DDB2*	*LIG1*		*BRCA1*	*MSH2*	
FANCE	*PRKDC*	*ERCC1*	*MBD4*		*BRCA2*	*MSH3*	
FANCF	*XRCC4*	*ERCC1*	*MPG*		*BRIP1*	*MSH4*	
FANCG	*XRCC5*	*ERCC2*	*MUTYH*		*FANCF*	*MSH5*	
FANCI	*XRCC6*	*ERCC3*	*NEIL1*		*MRE11A*	*MSH6*	
FANCL		*ERCC4*	*NEIL2*		*MUS81*	*PMS1*	
		ERCC5	*NEIL3*		*NBN*	*PMS2*	
		ERCC6	*NTHL1*		*POLQ*		
		ERCC8	*OGG1*		*RAD50*		
		POLE	*PARP1*		*RAD51B*		
		RAD23B	*PCNA*		*RAD51C*		
		RAD23B	*PNKP*		*RAD51D*		
		RFC1	*POLB*		*RAD52*		
		RFC4	*SMUG1*		*RAD54*		
		XAB2	*TDG*		*RBBP8*		
		XPC	*UNG*		*TOP3A*		
			XRCC1		*TP53BP1*		
					XRCC2		
					XRCC3		

Supplementary Table S2. The result of analysis using the signature analyzer based on MAF file

TCGA.Variant Caller.MAF	Number of Meta-Signature	MAX Cosine similarity					
		C>T CpG (COSMIC 1)	APOBEC (COSMIC 2)	APOBEC (COSMIC 13)	BRCA (COSMIC 3)	MSI (COSMIC 6)	COSMIC 10
**MuSE**	**5**	**0.93**	**0.85**	**0.81**	**0.86**	**0.82**	**0.98**
**MuTect**	**5**	**0.93**	**0.86**	**0.8**	**0.88**	**0.94**	**0.95**
**SomaticSniper**	**5**	**0.96**	**0.84**	**0.81**	**0.85**	**0.86**	**0.9**
**VarScan**	**5**	**0.95**	**0.85**	**0.81**	**0.83**	**0.85**	**0.96**
**Union**	**5**	**0.93**	**0.86**	**0.79**	**0.88**	**0.94**	**0.95**
**Intersect**	**5**	**0.94**	**0.85**	**0.81**	**0.86**	**0.83**	**0.97**
**Union.Del (TCGA-AN-A046)**	**4**	**0.93**	**0.85**	**0.81**	**0.88**	**0.84**	**-**
**Intersect.Del (TCGA-AN-A046)**	**4**	**0.94**	**0.85**	**0.81**	**0.86**	**0.83**	**-**

The first row shows the MAF file type; the second row shows the number of signature selected by the signature analyzer based on MAF file; the third row shows the Cosine similarity to the cosmic signature closest to the selected signature; the last two columns present the result values after removing the ultra-mutant sample (TCGA-AN-A046) that composes the singleton signature. In this study, intersect.del was used, after removing the ultra-mutant sample from the MAF file from which two or more common variants were obtained from calling based on the four types of TCGA maf.

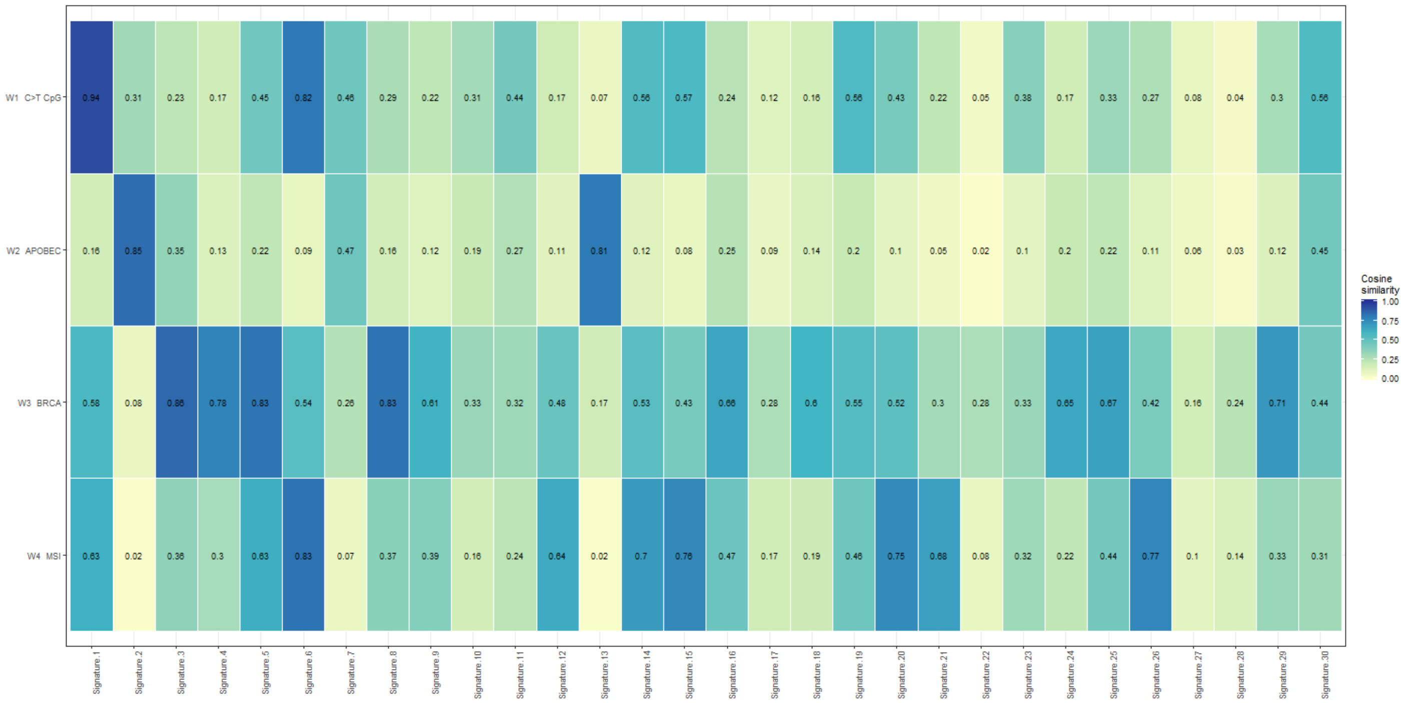

Supplementary Figure S1. Heat map of Cosine Similarity to meta-signatures obtained from signature analyzer with COSMIC signatures. The heat map shows Cosine similarity between the 20 cosmic signatures and Signature (W1 - 4) selected repeatedly in more than 40 rounds, by the 50th iteration of signature analyzer, based on the mutations collected from four variant caller types as they were called twice or more, and using Intersect.del.maf after removing the ultra-mutant sample (TCGA-AN-A046). Cosine similarity is expressed in values between 0 and 1, which was color-coded between light blue and dark blue.

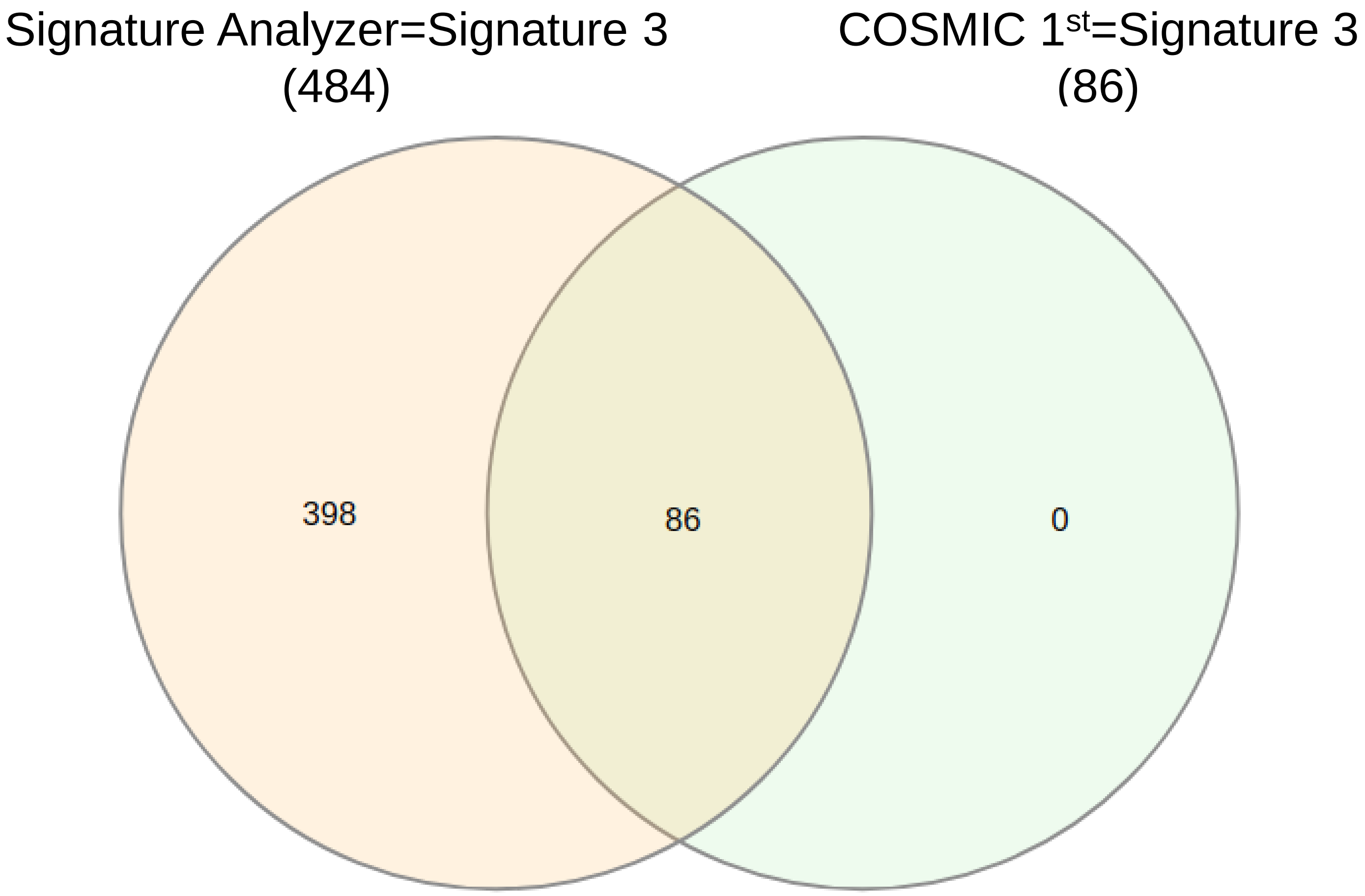

Supplementary Figure S2. Signature Analyzer-dominant patients include all COSMIC signature 3-dominant patients

We first counted patients whose signature 3-like result from Signature Analyzer was the most dominant. Then we compared this group to the patient group whose 1st ranking deconstructSigs-derived COSMIC signature was signature 3. All the latter patients were included in the former.

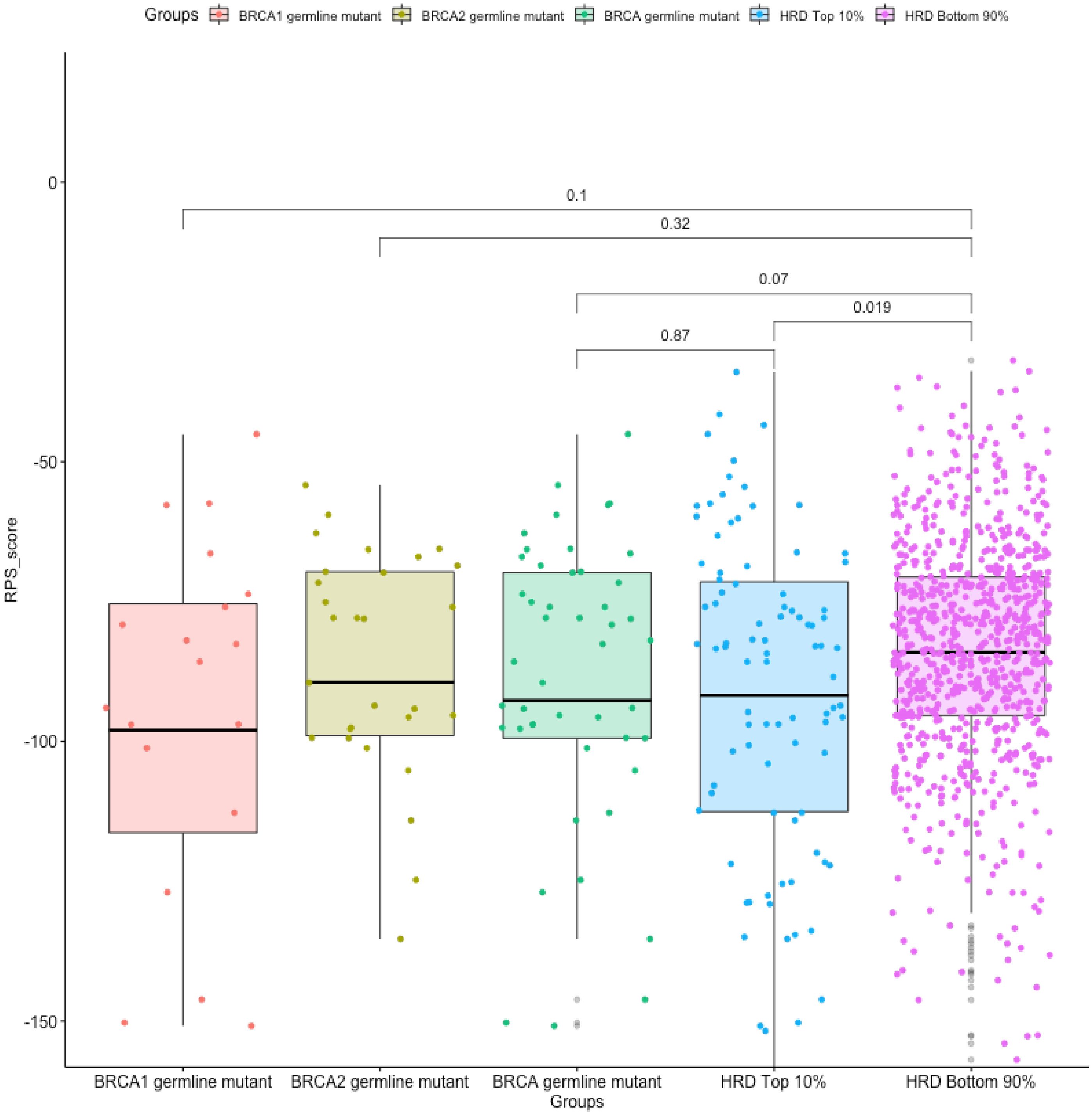

Supplementary Figure S3. Tumors with BRCA germline mutation or high HRD score show significantly low RPS score. Pairwise, the T test p-value for each group; the center line (bold) in the box plot shows the mean.

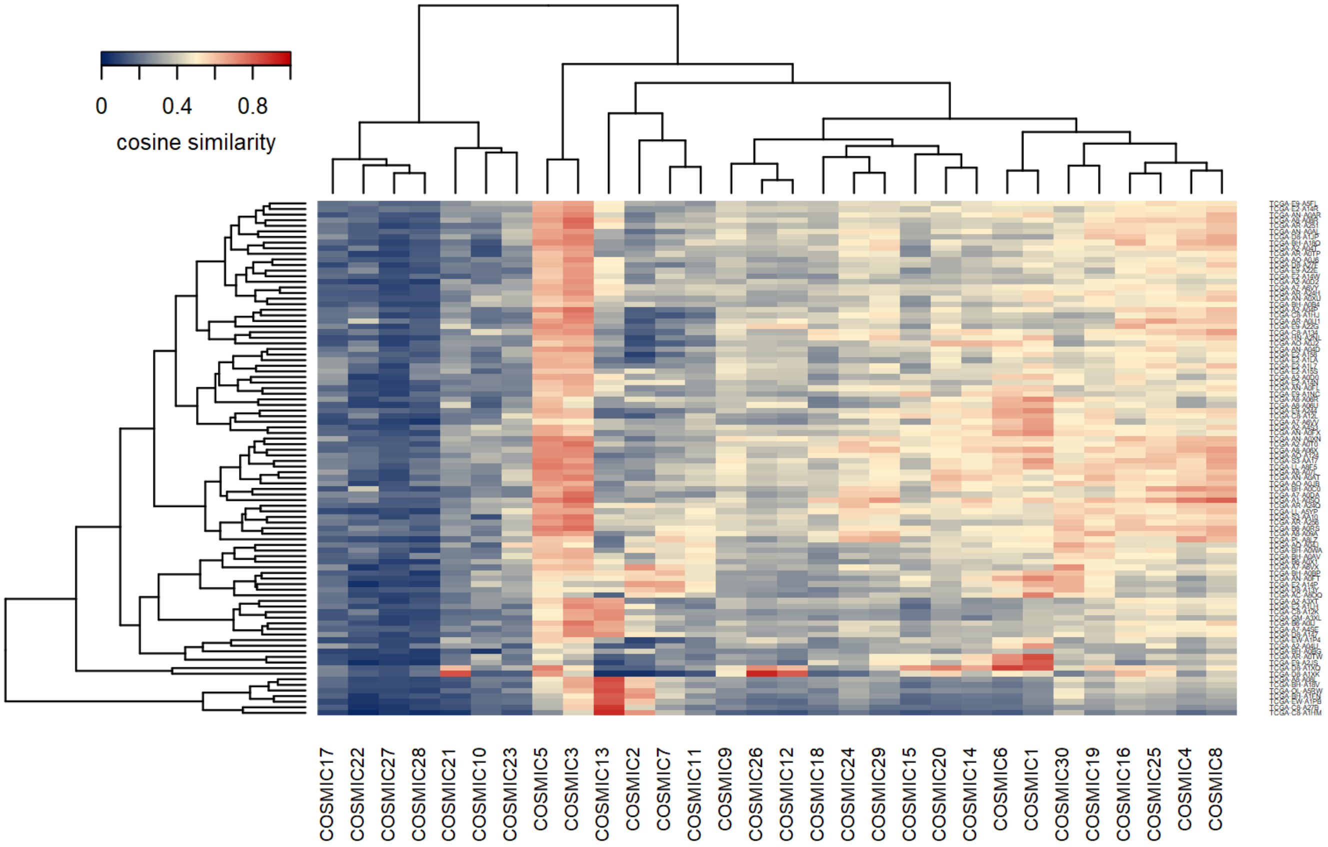

Supplementary Figure S4. Heat map of Cosine similarity between cosmic signature and 96 trinucleotide mutational pattern in HRD top 10 %. The trinucleotide mutational pattern was counted after categorizing the SNP and according to the 5’, 3’ nucleotides, and signature 3 and 5 were dominant in HRD top 10 % tumor.

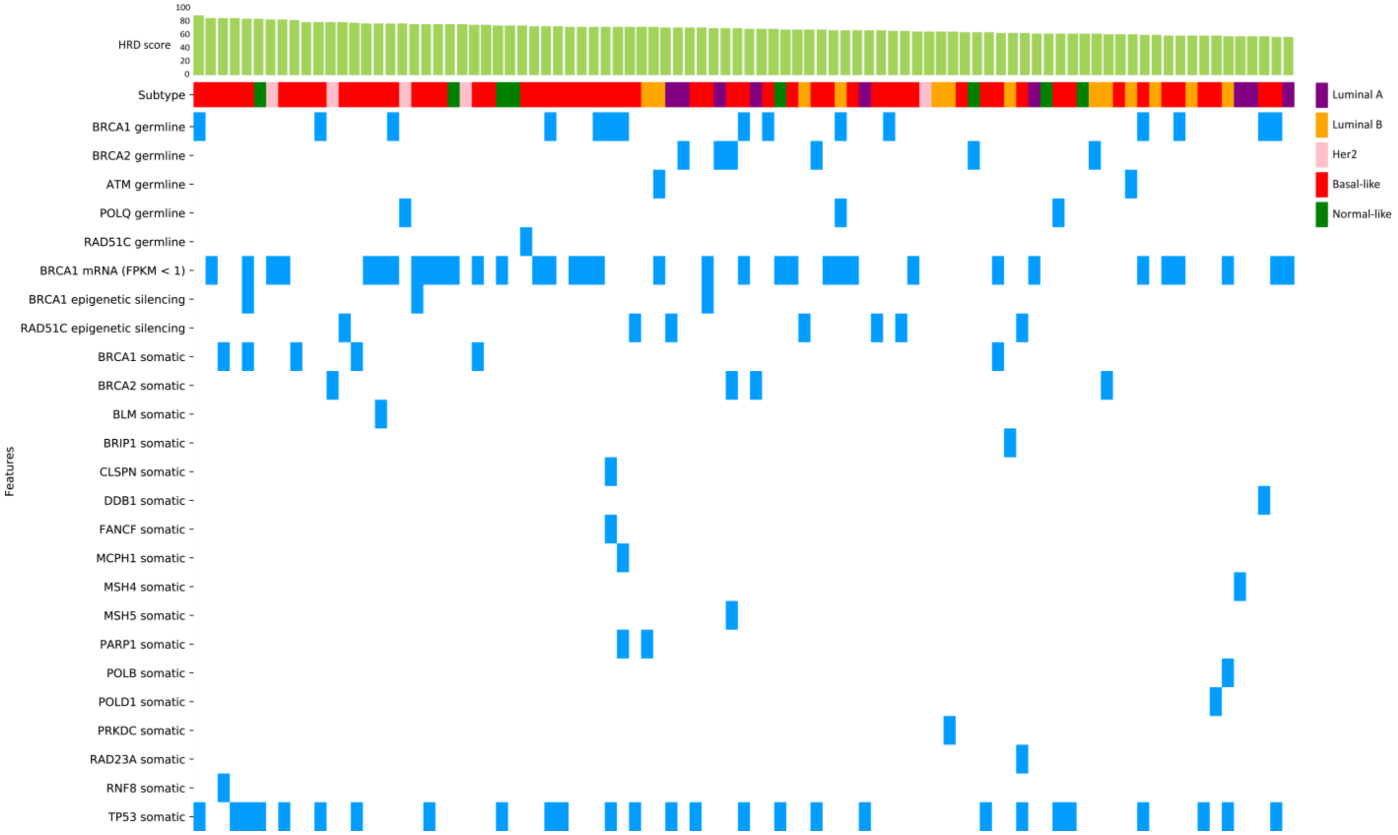

Supplementary Figure S5. Germline and somatic alteration feature of DDR gene in HRD top 10% tumor. The presence of germline mutation in 11 DDR genes; significant epigenetic silencing in the 12 genes; somatic alteration in 88 DDR genes, were assessed within HRD top 10 % tumor. Subtype color codes are as follows. Purple: luminal A, orange: luminal B, pink: Her2, red: basal-like, green: normal-like.

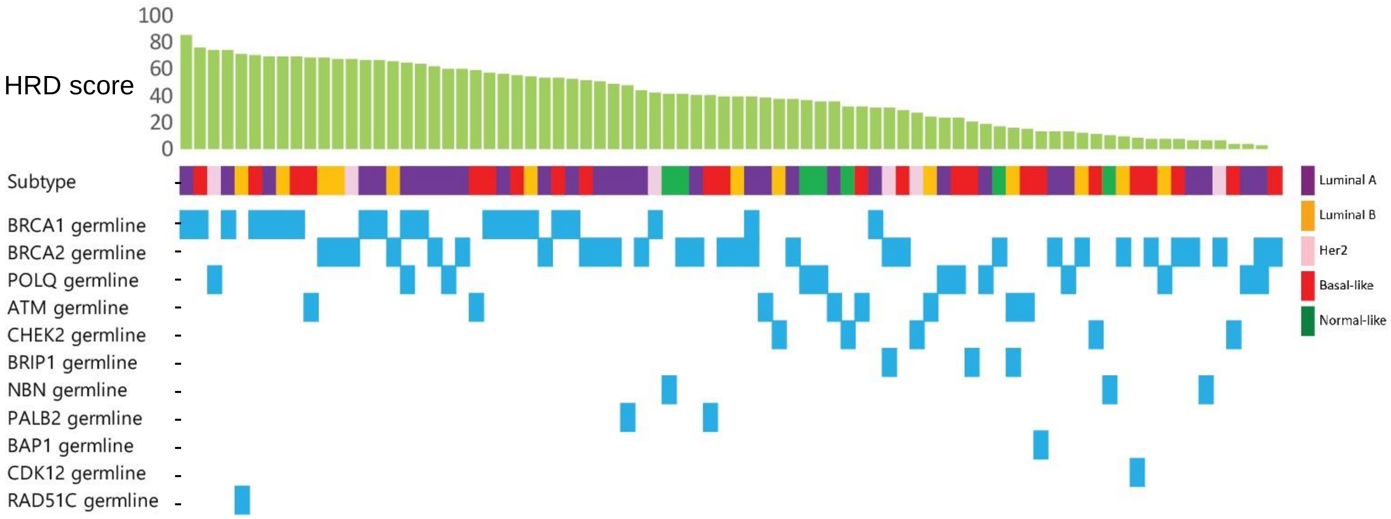

Supplementary Figure S6. Germline alterations of BRCA1/2 and other DDR genes in 81 tumors with DDR germline mutation. The presence of germline mutation in 11 DDR genes were assessed in tumors with DDR germline mutation including *BRCA1/2* regardless of HRD score. Subtype color codes are as follows. Purple: luminal A, orange: luminal B, pink: Her2, red: basal-like, green: normal-like.

A

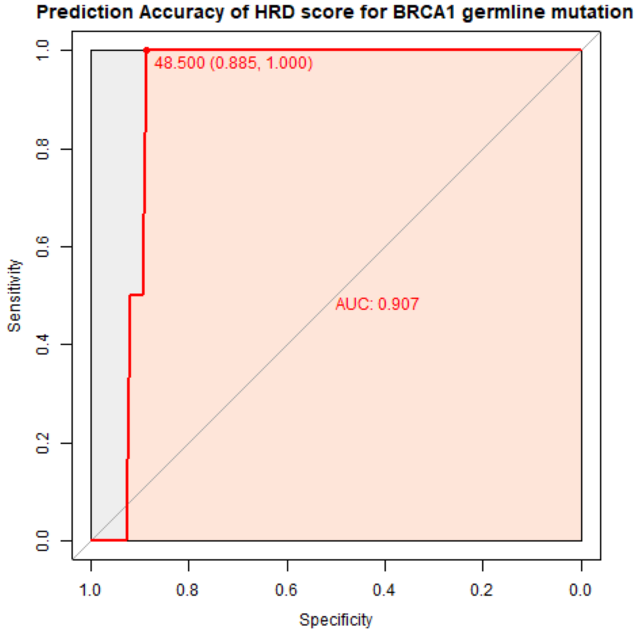

B

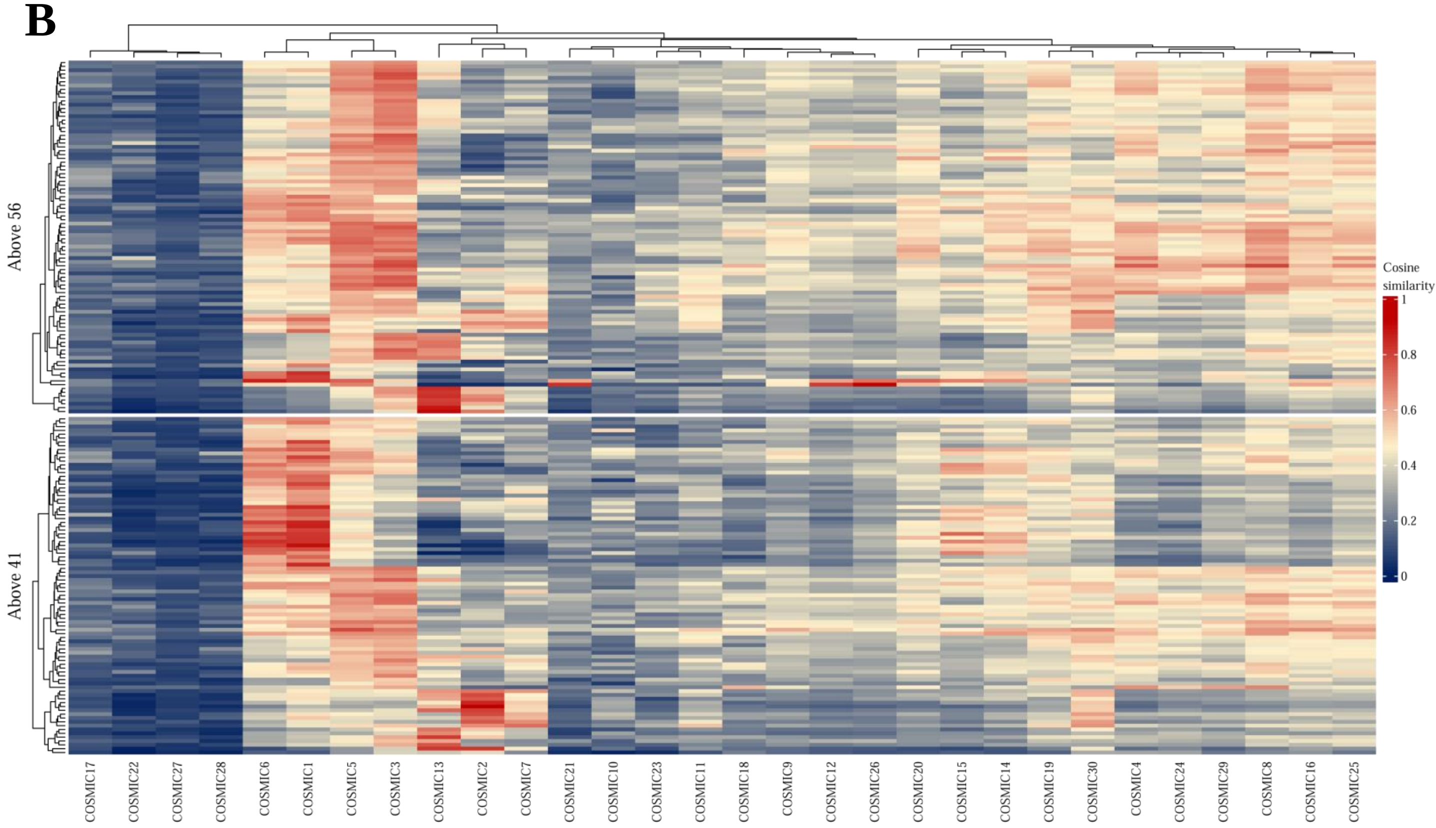

Supplementary Figure S7. The different cut-off of HRD scores for the definition of HRD tumor. A. ROC curve of the HRD score that best predicts BRCA germline mutation. B. Heat map of Cosine Similarity according to HRD scores.

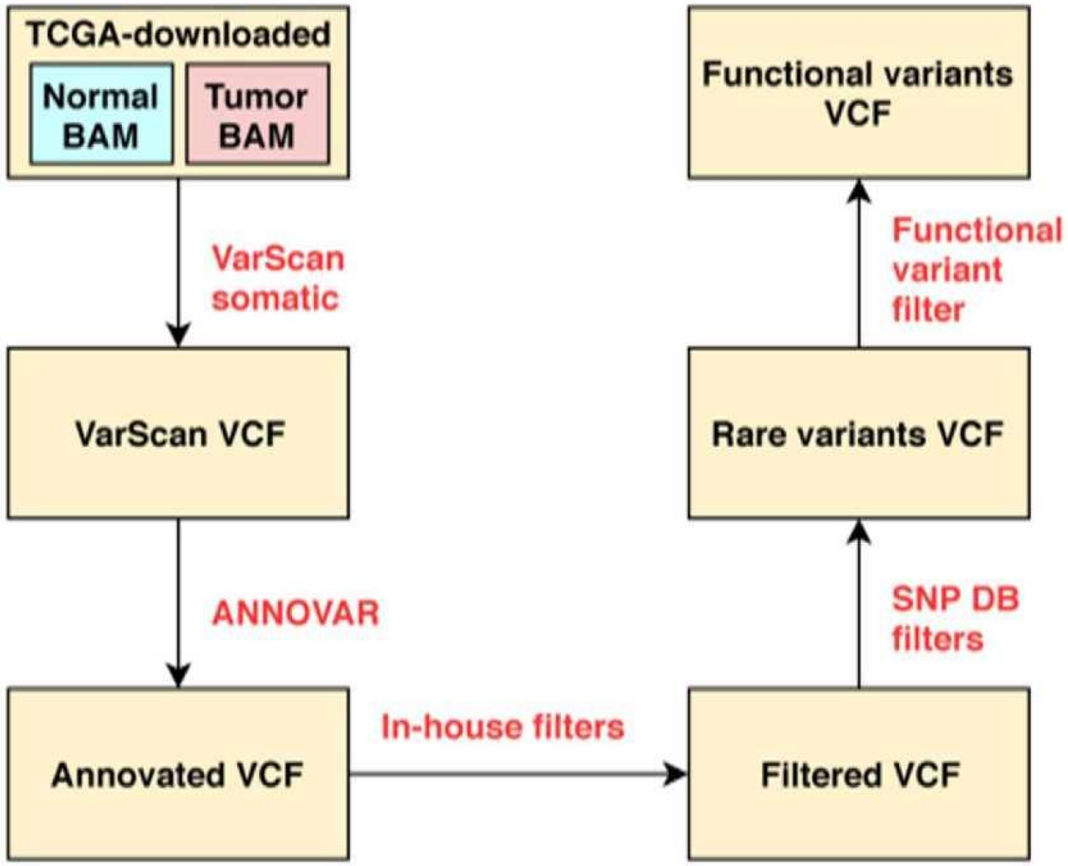

Supplementary Figure S8. Germline variant calling workflow diagram using raw TCGA alignment data.

The diagram shows how the germline variants were called from the raw TCGA alignment data. First, we performed VarScan2, and then annotated the variants using ANNOVAR, using default parameters for reference genome hg38. Next, we removed poor quality germline variants (with depth < 10, variant count < 5, variant allele fraction <0.08 or labeled as somatic), variants near homopolymer regions, and variants with an absolutedifference in mapping quality between reference and variant reads > 30, and variants positioned in read ends were removed using in-house filters. Then, common SNPs (variants with a minor allele frequency > 0.01 in either dbSNP or gnomAD) were removed. Only the variants that likely affect protein function were selected, by removing variants that are either intergenic, intronic, in UTR, or in ncRNA exons.
